# Formulation, Characterisation and Evaluation of the Antihypertensive Peptides, Isoleucine-Proline-Proline and Leucine-Lysine-Proline in Chitosan Nanoparticles Coated with Zein for Oral Drug Delivery

**DOI:** 10.3390/ijms231911160

**Published:** 2022-09-22

**Authors:** Minna Khalid Danish, John P. Gleeson, David J. Brayden, Hugh J. Byrne, Jesus M. Frías, Sinéad M. Ryan

**Affiliations:** 1 Environmental Science and Health Institute, Technological University Dublin, Grangegorman, D07 EWV4 Dublin, Ireland; 2 FOCAS Research Institute, Technological University Dublin, Camden Row, D07 EWV4 Dublin, Ireland; 3 UCD School of Veterinary Medicine, UCD Conway Institute, University College Dublin, Belfield, D04 V1W8 Dublin, Ireland

**Keywords:** Isoleucine-Proline-Proline (IPP), Leucine-Lysine-Proline (LKP), oral peptide delivery, zein, antihypertensive peptides

## Abstract

Isoleucine-Proline-Proline (IPP) and Leucine-Lysine-Proline (LKP) are food-derived tripeptides whose antihypertensive functions have been demonstrated in hypertensive rat models. However, peptides display low oral bioavailability due to poor intestinal epithelial permeability and instability. IPP and LKP were formulated into nanoparticles (NP) using chitosan (CL113) via ionotropic gelation and then coated with zein. Following addition of zein, a high encapsulation efficiency (EE) (>80%) was obtained for the NP. In simulated gastric fluid (SGF), 20% cumulative release of the peptides was achieved after 2 h, whereas in simulated intestinal fluid (SIF), ~90% cumulative release was observed after 6 h. Higher colloidal stability (39–41 mV) was observed for the coated NP compared to uncoated ones (30–35 mV). In vitro cytotoxicity studies showed no reduction in cellular viability of human intestinal epithelial Caco-2 and HepG2 liver cells upon exposure to NP and NP components. Administration of NP encapsulating IPP and LKP by oral gavage to spontaneously hypertensive rats (SHR) attenuated systolic blood pressure (SBP) for 8 h. This suggests that the NP provide appropriate release to achieve prolonged hypotensive effects in vivo. In conclusion, chitosan-zein nanoparticles (CZ NP) have potential as oral delivery system for the encapsulation of IPP and LKP.

## 1. Introduction

Hypertension is a serious medical condition that increases the risk of diseases of the heart, brain, kidneys and other organs [1]. Hypertension remains the most prevalent risk factor for cardiovascular diseases [2]. An estimated 1.28 billion adults aged 30–79 years worldwide have hypertension [1]. The main classes of antihypertensive therapies include diuretics, β adrenoceptor blockers (β-blockers), calcium channel blockers, angiotensin-converting enzyme (ACE) inhibitors, renin inhibitors, aldosterone antagonists and angiotensin II receptor blockers [3]. However, side effects have been reported with these treatments with chronic therapy.

ACE inhibitors are essential in the treatment for both high and low risk hypertension, and combination therapies such as “polypill” incorporate ACE inhibitors (enalapril) along with non-steroidal anti-inflammatory drugs (NSAID; aspirin), statins (atorvastatin) and diuretics (hydrochlorothiazide) [4,5]. Captopril, an ACE inhibitor, is a US Food Drug and Administration approved medication to manage primary hypertension. It was the first drug developed in this class [6]. Captopril has high oral bioavailability (~70%), is very effective in reducing systolic blood pressure (SBP) and reaches peak plasma levels within 45–60 min after oral administration [7]. However, captopril has a high incidence of side effects such as dry cough and acquired angioedema [8].

Proteins from food undergo enzymatic hydrolysis by digestive enzymes, thereby releasing smaller peptides, some of which have bioactive properties [9]. Some peptides inhibit ACE, and these can help maintain normal blood pressure and prevent escalation of hypertension by subverting the renin-angiotensin-aldosterone system [10]. Two such tripeptides are LKP, isolated from bonito fish and IPP, from milk β-casein following fermentation by *Lactobacillus helveticus* [11]. En route to the intestinal epithelium, the peptide must overcome several hurdles. These include instability at gastric and small intestinal pH values (pH 1–6), enzymatic degradation by pancreatic and brush border peptidases, passage across the mucus barrier, intestinal epithelial permeation, and degradation by liver first pass metabolism [12]. Both IPP and LKP have shown good stability in the gastrointestinal tract [13]. However, the bioavailability of both peptides is hampered by low permeation through the intestinal epithelium. To overcome this barrier, absorption enhancers and carrier systems, and device approaches have been used to enhance systemic uptake. Among these strategies, nanoparticle encapsulation is attractive due to the potential for enhanced uptake and sustained release [14].

Chitosan (CL113) is a non-toxic, biocompatible, linear polysaccharide that has shown promise for use in oral delivery systems, attributable to its ability to increase both mucoadhesion and epithelial permeability of bioactives [15,16]. Muco- and bio-adhesive properties of chitosan increase residence time within the gastrointestinal tract and assist the opening of epithelial tight junctions for paracellular transport [17,18]. Through the synthesis of CL113 into nano-based systems, encapsulation of bioactive macromolecules is achievable [19]. For example, CL113 nanoparticles have been shown to improve the oral delivery of encapsulated insulin in a rat model [20]. Several factors contribute to the complexation of two or more macromolecules and consequently, their optimal characteristics that render them applicable for oral delivery. For example, the degree of ionization, charge distribution, mass ratio and the temperature at which crosslinking takes place between both polyelectrolytes, have all been shown to have a significant effect on the resultant end product, such as nanoparticle size and surface properties [21,22].

A disadvantage of CL113-based nanoparticles is the inability to sustain release of an active following an initial burst release [23,24]. Additional outer surface coating can improve the integrity of the nanoparticles and control release profiles better. A popular approach to yield coated CL113 nanoparticles is by polyelectrolyte complexation, which exploits the interaction between positively charged CL113 and negatively charged polyelectrolytes such as alginate [25,26], dextran [27,28], hyaluronic acid [29,30] or zein [31,32]. Zein, a GRAS approved, prolamine-rich protein derived from maize [33], has potential advantages as a component of a nanocarrier for oral delivery systems due to its biocompatibility, low toxicity and capacity to increase permeation [34]. Zein also improves entrapment of bioactives, in addition to sustaining the release profile by providing protection against harsh conditions in the gastrointestinal tract [35,36].

In this study, CL113 nanoparticles coated with zein were investigated as a potential oral delivery system for LKP and IPP. The formulation parameters were systematically studied for both IPP and LKP loaded nanoparticles. The optimised nanoparticles were assessed for ACE inhibition, stability after freeze drying and then characterised both in vitro assays and following oral gavage in an SHR model.

## 2. Results

### 2.1. Physicochemical Characterisation of Peptide Loaded Chitosan (CL113 NP) and Chitosan Zein Nanoparticles (CZ NP)

The optimum encapsulation achieved for CL113 nanoparticles, loaded with either LKP or IPP were at a mass ratio of 6:1 and 4:1 (CL113: TPP), as summarised in Table 1.

To increase the encapsulation efficiency (EE%) of LKP and IPP, zein was added to the formulation mixture, to yield CZ NP. Ratios of CL113: zein were formulated (1:0, 1:0.5, 1:1, 1:2) and the EE%, particle size and ZP were measured. A concentration of 100 µg/mL of peptide was chosen to identify more optimal concentrations and interaction of zein and CL113 with the peptides. A ratio of 1:1 (CL113: zein) produced an EE > 80% with satisfactory physicochemical characteristics for LKP (301 ± 16 nm, 41 ± 1 mV) and IPP (223 ± 9 nm, 46 ± 2 mV), as shown in Table 2.

**Figure 1:**
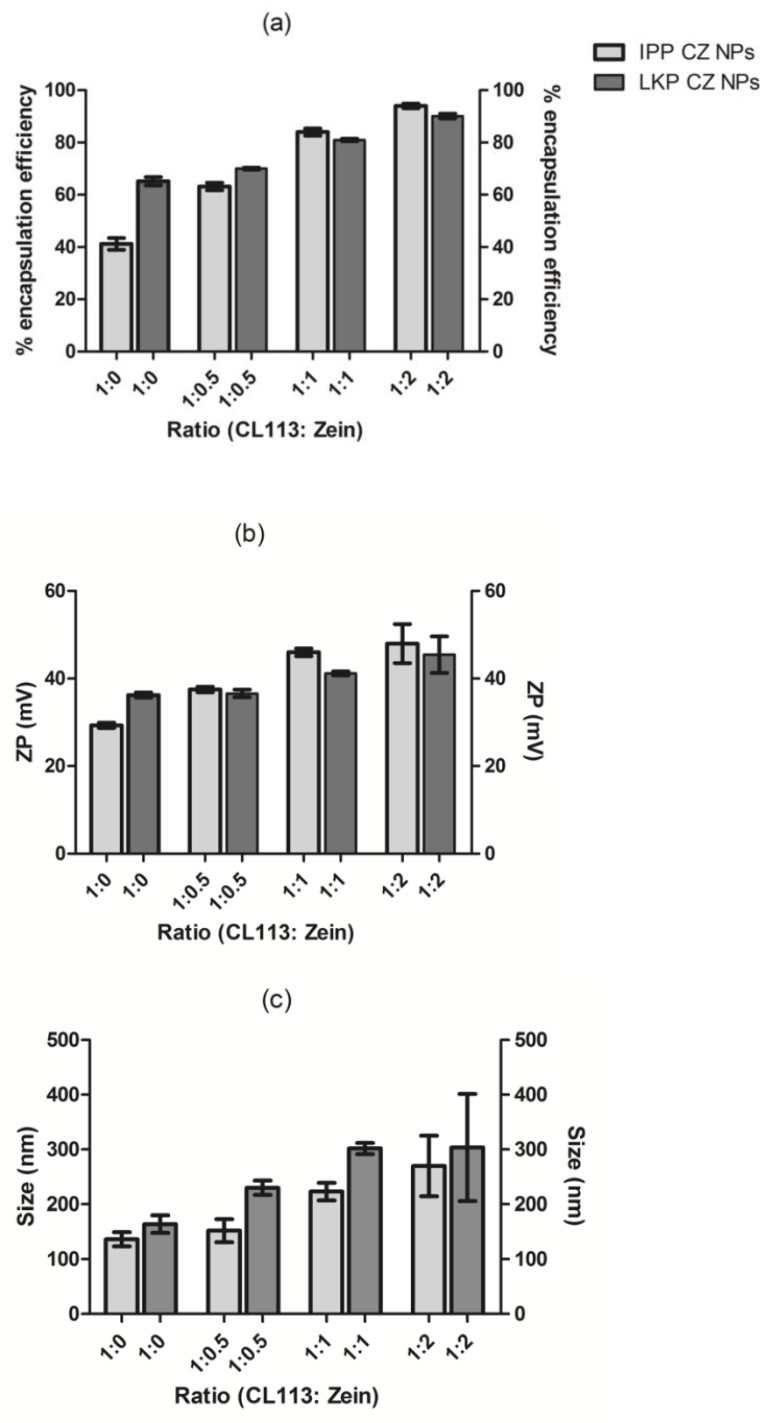
The effect of (**a**) EE (%), (**b**) ZP (mV) and (**c**) size (nm) at varying ratio of CL113: zein (from 1:0 to 1:2) for CZ NP. Data are expressed as a mean ± SD of n = 3.

IPP had the best interaction with zein, increasing its EE from 41 to 63 to 84% encapsulation with an increase in CL113: zein (1:0 to 1:0.5 to 1:1), respectively. The addition of zein increased the loading capacity (LC) by 4 and 5 fold for LKP and IPP, respectively. LKP’s EE was increased by only 10% with zein at a 1:1 ratio. A further increase was observed for a ratio of 1:2 (CL113: zein); up to 90% for LKP and 95% for IPP encapsulation, but variable physicochemical characteristics were obtained at this ratio (Figure 1). A 1:1 ratio was therefore selected for further in vitro and in vivo studies.

**Figure 2:**
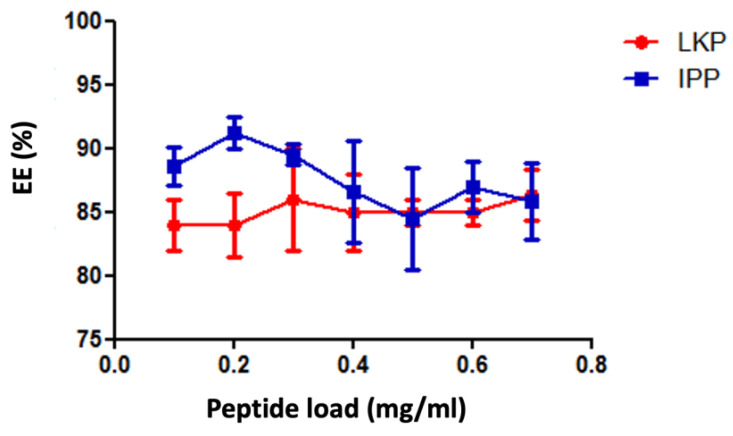
Changes in EE% for CZ NP loaded at different concentrations of IPP and LKP. Data are expressed as a mean ± SD of n = 3 batches.

The peptide loading concentration was then further investigated at a ratio 1:1 (CL113: zein) to ensure that a sufficiently high therapeutic dose could be delivered via the oral route. Consequently, peptide concentrations from 100–700 µg/mL were added to the formulation (20 mL final volume of formulation) during the cross-linking process and the EE% was determined. The results are presented in Figure 2, which demonstrates that no significant changes in EE% were observed over varying peptide concentrations for both IPP and LKP. However, the least variance was observed for formulations loaded at 600 µg/mL, and therefore this concentration was selected for further studies.

### 2.2. Chemical Characterisation of CZ NPs Using FTIR Spectroscopy

**Figure 3:**
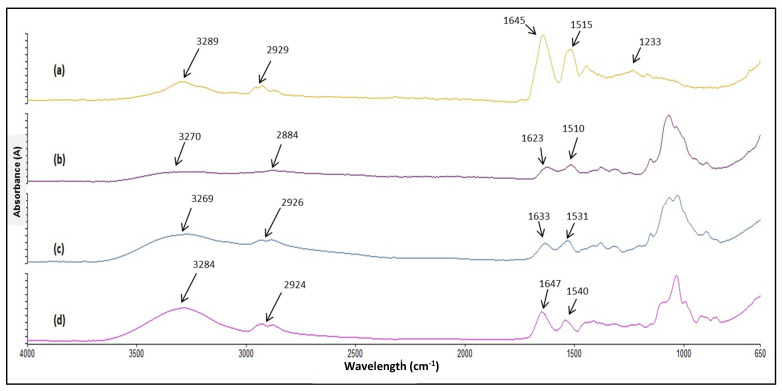
FTIR spectra of (**a**) zein, (**b**) CL113, (**c**) LKP NP and (**d**) LKP CZ NP.

FTIR spectra of zein, CL113, LKP NP and LKP CZ NP are shown in Figure 3, with the chemical features relevant to the nanoparticle formation and interaction highlighted. No significant spectral changes were observed between LKP or IPP nanoparticles, and hence the spectra of only LKP-loaded NP are presented in Figure 3.

A strong increase in absorbance was observed for the bands at 3269, 2926 cm^−1^ for LKP NP (c) when compared to CL113 (b), indicating that hydrogen bonding between TPP, chitosan and the bioactive has occurred, facilitating the formation of nanoparticles. The absorbance of the high frequency band (3284 cm^−1^) was seen to increase twofold in zein coated NP (d), which may be attributable to intermolecular interactions between CL113 and zein, formed between amide groups of glutamine in zein and both amide and hydroxyl groups of CL113 [37]. The band at 1645 cm^−1^ of the zein (a) is attributed to the α-helical component of the secondary structure of zein, which is also present in the CZ-NP at 1647 cm^−1^, indicating that the α-helical structure of zein is most likely preserved after encapsulation [38,39]. Frequency shifts observed in the 1515 cm^−1^ feature in the zein spectrum (a) to 1540 cm^−1^ in the CZ NP (d) may be due to the amino group interaction between zein and CL113 chains [37]. In addition, the characteristic peak at 1233 cm^−1^ in zein was not visible in the CZ NP. This may be a result of C-N stretching and N-H place deformations [40].

**Figure 4:**
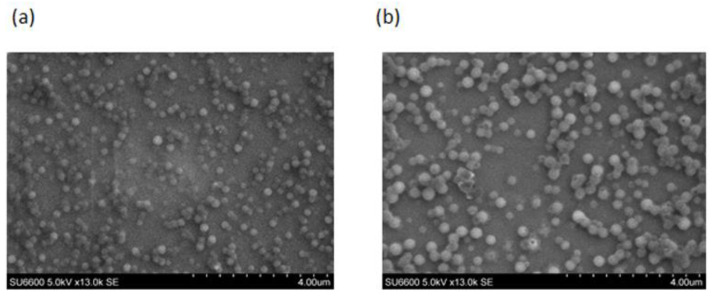
SEM images of (**a**) IPP-CZ NP and (**b**) LKP-CZ NP.

For SEM analysis (Figure 4), similar morphologies were observed for both LKP and IPP entrapped CZ NP, whereby, spheroidal nanoparticles were formed. According to both SEM and DLS analysis, particle sizes increased after the addition of zein. LKP NP increased in size from 140 ± 10 nm to 330 ± 20 nm (LKP-CZ NP) and IPP NP from 111 ± 30 nm to 249 ± 10 nm (IPP-CZ NP).

### 2.3. ACE Inhibition of CZ NP

The ACE inhibition IC_50_ for captopril, LKP and IPP, before formulation into NP, were determined to be 0.006 ± 0.002 µM, 0.36 ± 0.01 µM and 3.1 ± 0.6 µM, respectively. The bioactivity assessment was carried out for the free peptides, unloaded NP, peptide encapsulating NP and CZ NP to identify any potential interference or denaturation that may have occurred during the formulation process. The IPP/LKP CZ NP presented in Table 2 were selected for bioactivity analysis. Since no leakage of the peptides was observed after the ultrafiltration-centrifugation, the CZ NP were subjected to sonication to release the peptides and to determine the ACE inhibition activity. Table 3 presents the % ACE inhibition for Cl113 NP, and CZ NP formulations diluted to contain a peptide concentration of 10 µM. From the bioactivity assay analysis, both IPP and LKP maintained their bioactivity as ACE inhibitors after the formulation process.

### 2.4. Stability of CZ NP during Lyophilisation

**Figure 5:**
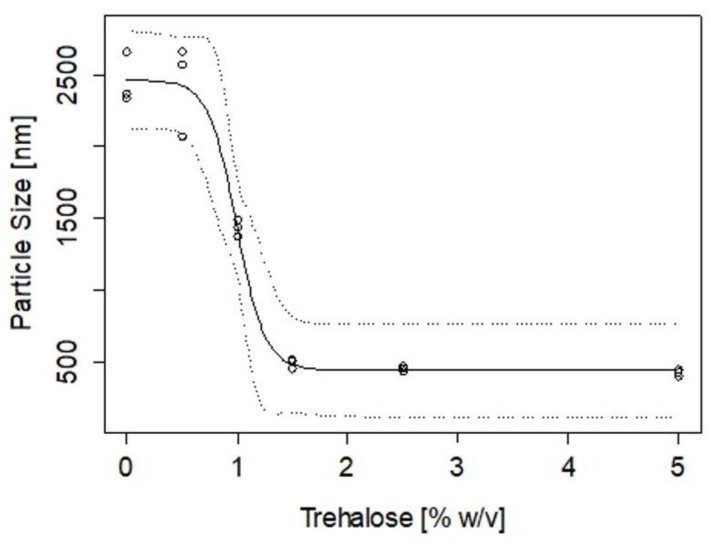
Particle size differences of freeze-dried nanoparticles after resuspension with a logistic model fit where the top and bottom dash lines represent the 95% confidence coverage.

The change and rate of aggregation of nanoparticles were assessed after lyophilisation using trehalose. Concentrations up to 5% *w*/*v* were added to the CZ NP (without peptide) and the particle sizes and zeta potentials were measured after lyophilisation. Using a logistic model (Equation (3)), results for particle size showed >1.5% *w*/*v* of trehalose was needed to maintain the integrity of the nanoparticle in its final state prior to oral administration (Figure 5).

**Figure 6:**
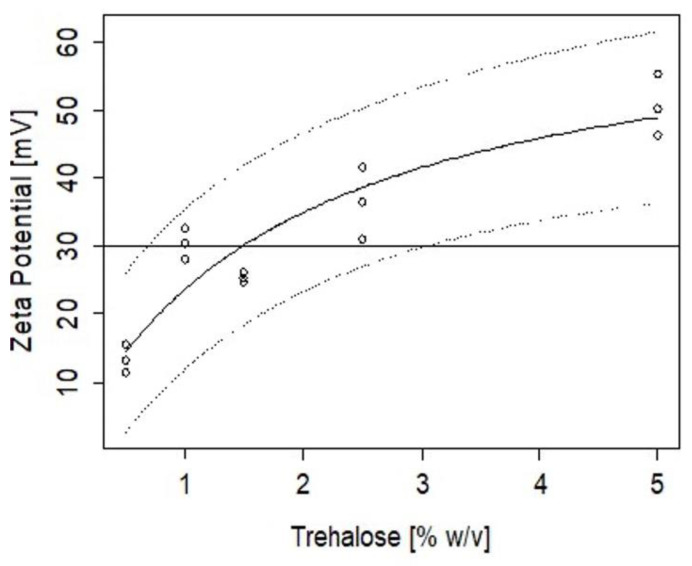
Zeta potential variations with added trehalose (% *w*/*v*) where the top and bottom dash lines represent the 95% confidence coverage.

The inverse prediction of the logistic model fit to the data in Figure 5 indicates that the minimum amount of trehalose that would ensure a particle size below 500 nm is 1.4 ± 0.4%, with an upper limit of 2.27% that would warrant 95% confidence. Similarly, Figure 6 shows the change in ZP as a function of the % of trehalose added. The inverse prediction to the Michaelis-Menten model applied to the experimental data indicates that to achieve a formulation of nanoparticles with ZP of 30 mV, a minimum trehalose concentration of 1.5% is required, with an upper concentration of 2.54% to warrant 95% confidence in this condition.

### 2.5. Cytotoxicity Assessment of Peptide CZ NP

**Figure 7:**
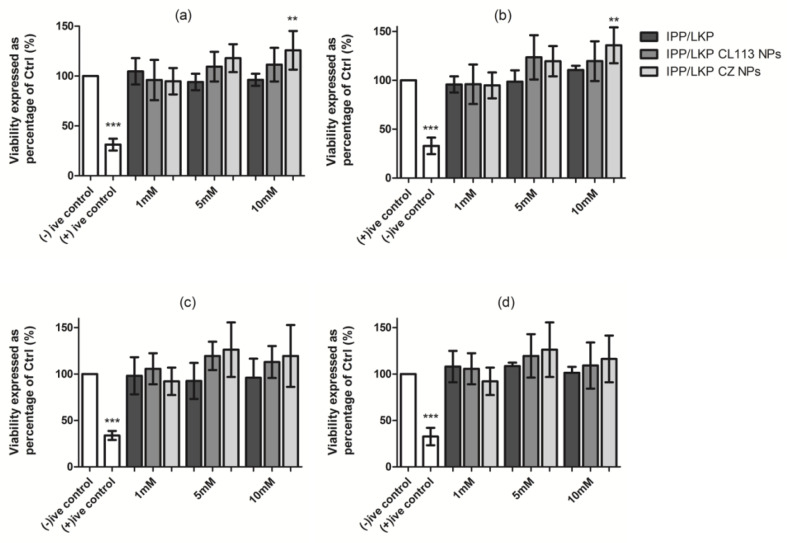
Comparison of cytotoxicity of native LKP, LKP NP and LKP CZ NP exposed to (**a**) Caco-2 cells lines (24 h) and (**b**) HepG2 cell lines (72 h). Native IPP, IPP NP and IPP CZ NP exposed to (**c**) Caco-2 cells lines (24 h) and (**d**) HepG2 cell lines (72 h). Triton X (0.05% *w*/*v*) and 100% cell viability (no treatment) were used as the positive and negative control. One-Way ANOVA with Dunnett’s post-test *** *p* < 0.001, ** *p* < 0.01. Each value represents the mean ± SD, n = 3 independent experiments for each concentration and time point with replicates of three.

The MTS assay was used to evaluate the cytotoxicity of CZ NP in Caco-2 and HepG2 cells. There was no evidence of cytotoxicity for either the free peptides or peptide loaded NP (Figure 7) at concentrations from 1 to 10 mM at 24 h exposure for Caco-2 cells and 72 h for HepG2 cells. The cell viability for loaded CZ NP (both IPP and LKP) was over 70% at concentrations of the peptide and peptide NP from 1 to 10 mM. Prior to exposure of Caco-2 and HepG2 cell lines, NP were subjected to sonication (5 s on/off pulse for 30 s) to avoid agglomeration. An increase in proliferation of cells was observed for CZ NP test samples at 5 and 10 mM for each peptide (Figure 7).

### 2.6. In Vitro Controlled Release Studies of CZ NP

**Figure 8:**
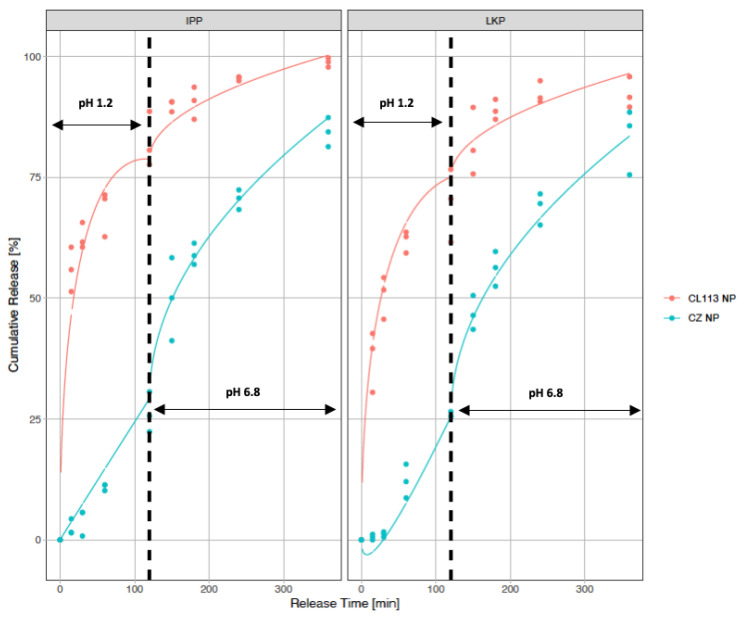
Release kinetics of IPP/LKP NP and IPP/LKP CZ NP. Data is expressed as n = 3.

The in vitro release profile of LKP and IPP CZ NP was measured over 6 h in total (2 h in SGF pH 1.2, followed by 4 h in SIF pH 6.8). Uncoated peptide entrapped NP were used as the control, as illustrated in Figure 8. Both LKP and IPP release profiles displayed an initial burst (>50% release) without zein in SGF (pH 1.2), with LKP exhibiting a 10% slower burst compared to IPP. During the first two hours of the release process, both peptides showed a diffusion and/or erosion from the NP into the medium (SGF). After 2 h (120 min), a decrease in release rate is observed, i.e., approaching zero-order kinetics. With the addition of zein however, less than 20% (compared to 70% without zein) cumulative release occurred for each peptide in SGF over 2 h, followed by a sustained release in SIF over 6 h. At the end of this process over 85% of peptide was released (Figure 8).

To further understand the peptide release mechanism, Table 4 presents the parameters for LKP and IPP release in SGF (*ks*) and SIF (*ki*). The rate constants represent two distinct processes; *ks*_1_ which represents a diffusion mechanism and *ks*_2_ representing a relaxation mechanism with an R^2^ of 0.985. IPP and LKP CL113 NP showed a significant difference in *ks*_1_ and, in terms of *ks*_2_, IPP CL113 NP exhibited a faster release. For IPP and LKP CZ NP, *ks*_1_ was not significant and a zero order (*ks*_2_) response was observed in SGF. In SIF, a Higuchi release phenomenon was observed (*ki*_2_ was not significant). Significant coefficients are listed in Table 4. Both IPP and LKP CZ NP had the same rate constants for *ks*_2_ and *ki*_1_ indicating that the release was not dependent on the peptide once zein was incorporated into the formulation.

### 2.7. Pharmacodynamic Studies of Oral Administered Zein/Chitosan Loaded Nanoparticles Using the SHR Model

**Figure 9:**
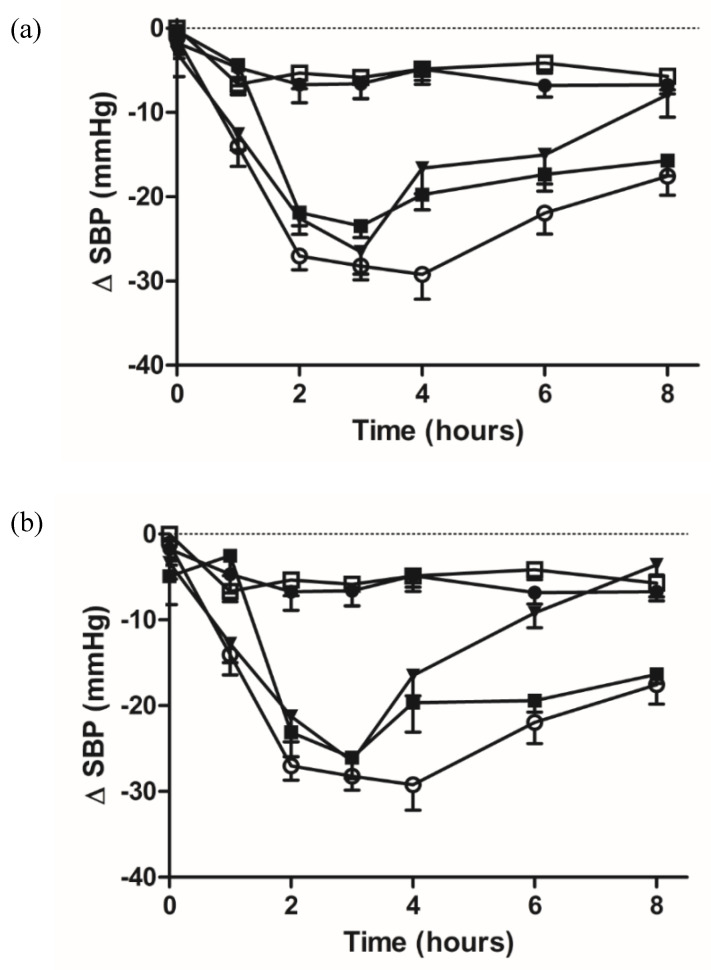
Change in SBP after oral administration to SHRs. (**a**) IPP and (**b**) LKP in free or entrapped format. Captopril (5 mg/kg; ○) and DPBS (●) controls. Single oral gavage administrations were performed with a dose of 10 mg/kg of peptide (encapsulated ■ or free ▼) and unloaded NP (□). Each value represents the mean ± SEM, n = 6 independent animals.

To investigate the potential blood pressure lowering effect of NP encapsulated IPP and LKP in vivo, SHRs were dosed by oral gavage (IPP and LKP 10 mg/kg, IPP CZ NP and LKP CZ NP 10 mg/kg of peptide) and SBP was recorded (Figure 9). Captopril (5 mg/kg) was used as the positive control, while DPBS and unloaded NP were the negative controls. Both free peptides lowered SBP, however captopril and the NP entrapped peptides had a longer sustained attenuation of SBP (Table 5). The pharmacodynamic (PD) effect initiated between 1 and 2 h after oral administration of both free and NP entrapped tripeptides. The in vitro release data suggest that the NP encapsulated peptides are slowly released over 4–6 h, resulting in prolonged exposure time for intestinal uptake. This results in a sustained attenuation of SBP up to 8 h.

## 3. Discussion

Particle size, polydispersity index (PDI) and ZP are important parameters for NP characterisation and optimisation [41]. Optimal characteristics for oral delivery systems have been reported to have particle size ranging 100–500 nm [42], PDI < 0.4 [43] and ZP > 30 mV [43,44]. The route of administration is one of the critical factors that govern the formulation of a bioactive. For oral peptide delivery, intestinal permeation, luminal, brush border and cytosolic metabolism, and hepatic clearance mechanism, are all typical limiting factors for poor bioavailability [45,46]. It has previously been reported that IPP and LKP are stable in low pH, intestinal and liver peptidase, indicating their ability to withstand first pass metabolism [13]. Taking this into account, the main limiting factor is for the NP to release the peptides close to the intestinal wall in high concentration to facilitate permeation and uptake across the epithelia. Gleeson et al. showed that both IPP and LKP are highly permeable and cross small intestinal epithelia in part by the PepT1 transporter and via the paracellular route in the small intestine [47]. Formulation of LKP and IPP into a nanoparticle composed of CL113 showed good overall physicochemical characteristics but were unsuitable for oral delivery under physiological conditions due to undesirable early burst release. To overcome this, zein was introduced as a secondary coating component via polyelectrolyte complexation to the peptide encapsulated NP. Mass ratio 1:1 (CL113: zein) produced NPs with the highest encapsulation efficiency whilst maintaining optimal particle sizes, ZP and PDI required for oral delivery. The encapsulation efficiency of IPP significantly increased from 41% to 84%, after the addition of zein to the formulation mixture. A possible explanation could be that IPP is composed of two proline residues which provided a more hydrophobic environment for both protein and peptide to interact. Vozza et al. reported that encapsulation efficiencies of 80.7% and 78.9% were similarly achieved, respectively, for methyl selenocysteine and selenocysteine loaded chitosan NP [31]. Karthikeyan et al. previously reported that zein and hydrophobic drugs performed better during the development of sustained drug delivery systems. They found that the hydrophobic drug aceclofenac, encapsulated with zein showed stronger interactions, smaller sizes and a more prominent sustained release profile compared to hydrophilic compounds [48]. Lai et al. encapsulated 5-fluorouracil into zein nanoparticles for liver targeting in a mouse model. The optimised formulation had an encapsulation efficiency of up to 60% with sustained release behaviour [49]. Zein nanoparticles have also been used as a platform in combination chemotherapeutics for the delivery of vorinostat and bortezomib in prostate cancer. These nanoparticles exhibited an entrapment efficiency above 60% combined with controlled release behaviour. The system showed higher release under acidic compared to basic conditions due to lower stability and a conformational change in the zein structure in an acidic pH [50].

Furthermore, both the IPP and LKP loadings were increased in CZ NP. Between 100 and 700 µg of peptide was encapsulated into the CZ NP, for which 600 µg provided the best physicochemical characteristics for oral delivery with high EE (>85%), particles sizes (200–400 nm) and ZP (39–41 mV).

Zein and chitosan have isoelectric points of 6.2 and 6.8 [51,52], respectively, which may result in poor physical stability and dispersibility in neutral pH in aqueous systems, limiting the potential to produce efficient delivery systems. Zein is amphiphilic and has hydrophobic regions that degrade slowly by hydrolysis but can cause aggregation into colloidal particles [53,54]. A number of stabilisers have previously been used to help reduce aggregation of NP during lyophilisation, for example trehalose [55,56], sodium caseinate [57], lecithin and Pluronic F68 [58]. Trehalose was used for this study to replace water molecules around the CZ NP and to retain their spatial structure via bulk hydrogen bond interactions [52]. Our findings showed that a minimum 2.3% trehalose was required to allow re-suspension of CZ NP in aqueous conditions.

When developing an oral delivery system, it is important to investigate interactions of the bioactive peptide with the other excipients in the formulation. During the formulation process, peptides are exposed to several different conditions, for example changes in pH, ionic strength, and polymer interaction. Therefore, the efficacy of ACE inhibition after entrapment in NP was assessed to ensure that the peptides will be bioactive following administration. The IC_50_ of IPP and LKP has been previously reported. Fujita et al. reported the IC_50_ value of LKP as 0.32 μM [59]. Different IC_50_ values have been reported for IPP, varying from 1.89 to 5 μM [60,61]. Loaded NP were subjected to sonication to allow the peptides to be released. Results showed no significant reduction in ACE inhibition induced by NP released peptides compared to free peptides, confirming that the peptides remained bioactive, and that no denaturation occurred during the formulation process.

Cytotoxicity assessment using the MTS assay of CZ NP in Caco-2 and HepG2 cells showed no decrease in cell viability. A previous study also showed no cytotoxicity or clastogenicity in Chinese hamster lung cells for IPP and VPP [62]. In another study, both the MTS and High Content Analysis confirmed no potential for either IPP or LKP to induce cytotoxicity at 1, 5, 10 mM concentrations [13]. This is in agreement with the findings of this current study. Significant proliferation was observed for Caco-2 cell lines when exposed to high concentrations of CZ NP (>5 mM). A possible explanation could be that the surface hydrophobicity of zein in the CZ NP may result in high adhesion of the NP to cells [63]. Casey et al. reported the interference of the nanomaterials with the cytotoxicity assay subsequently showing false positives within results. They reported that nanotubes interfered with absorption/fluorescent emission spectra [64]. 

The in vitro sustained release of IPP and LKP from CZ NP was pH dependent. NP were exposed to SGF for 2 h followed by 4 h in SIF to help mimic the oral delivery route using a dialysis membrane technique. This profile was modelled using a swelling Peppas equation (Equations (4) and (5)). The release of LKP and IPP from the nanoparticles is thus proposed to be due to a combination of diffusion and relaxation mechanisms. The addition of zein resulted in an increase in the release profile in pH 1.2 (SGF), with only 20% of LKP and IPP being released (compared to 70% without zein) via a zero-order mechanism. This was followed by a Higuchi diffusive profile after 4 h in pH 6.8, by which 85% of LKP and IPP were released (Figure 8). This result agrees with Penalva et al., who reported a diffusion and zero order kinetic profile for resveratrol from zein NP [65].

The in vitro studies of encapsulated IPP/LKP, showed good stability and a sustained controlled release of both tripeptides. A similar effect was observed when both IPP and LKP CZ NP were administered by oral gavage in the SHR rat model. Captopril is an oral antihypertensive drug which has as a single oral dose effective for 6–8 h. Clinical use requires a dose of 37–75 mg of captopril to be taken three times daily [4]. Hence, LKP/IPP CZ NP were tested over 8 h. The results presented for free IPP and LKP showed a reduction in blood pressure for only the first 3 h of administration. However, when encapsulated with CZ NP a sustained release was observed with a reduction in blood pressure up to 8 h, similar to captopril. Inchaurraga et al. also utilised zein to formulate insulin nanocarriers coated with a poly(anhydride)-thiamine conjugate. Their findings showed the nanoparticles decreased the blood glucose levels in diabetic rats at a dose of 50 IU/kg up to 20% of their basal glucose levels. The hypoglycemic effect was also observed during 18 h [66]. Overall, the significant improvement in terms of pharmacological activity and relative bioavailability of both IPP and LKP may be partly explained by the sustained controlled release and the protection effect of the nanoparticle shield provided by zein.

## 4. Materials and Methods

### 4.1. Materials

LKP (Mw 356.47, purity = 97% according to the manufacturer’s specifications) and IPP (Mw 325.41, purity = 97% according to the manufacturer’s specifications) were synthesised by ChinaPeptides Co. Ltd., (Shanghai, China). Chitosan (CL113, Mw = 110 kDa, deacetylation degree = 86% according to manufacturer’s specifications) was obtained from Pronova Biopolymer (Oslo, Norway). Zein, purified, was purchased from ACROS Organics™, Fisher Scientific. CellTitre 96^®^ AQ_ueous_ One Solution Cell Proliferation Assay was supplied by Promega (Madison, WI, USA). Caco-2 cells (passage 26–32) were obtained from the European Collection of Cell Cultures (Salisbury, UK). HepG2 (34–40) cells were obtained from the American Type Culture Collection. Ultrapure water was used for all experiments and was obtained from a Milli-Q water purification system by Millipore (Darmstadt, Germany). TPP (sodium tripolyphosphate) and all other reagents were purchased from Sigma Aldrich, Dublin, Ireland.

### 4.2. Formulation of Peptide Loaded Chitosan Nanoparticles

Preparation of peptide loaded NP was based on a modified ionotropic method [67,68]. The optimal concentrations of CL113, a crosslinker, TPP and tripeptides IPP/LKP for the formation of NP is indicated in Table 1. Stock solutions of 10 mg/mL CLL113 and TPP were prepared. CL113 was dispersed in acetate buffer (pH 3) and TPP in 0.01 M sodium hydroxide solution. The stock solutions of CL113 and TPP were diluted to different concentration ratios at a fixed volume mixture of 2.5:1 CL113: TPP containing solution. A fixed concentration of 0.1 mg/mL LKP was added to the diluted TPP solutions. The TPP-peptide solution was added dropwise to the CL113 solution while stirring at 800 rpm for 30 min. NP were then coated with zein or isolated using ultracentrifugation (Centriplus YM-30, MWCO of 30 kDa, Millipore, Darmstadt, Germany) at 3000 rpm for 30 min and resuspended in deionised water for physicochemical analysis.

### 4.3. Coating of Peptide Loaded Chitosan Nanoparticles with Zein

After the LKP/IPP CL113 NP had stabilised for 30 min, 8 mL of absolute ethanol was added dropwise to the formulation and stirred for 30 min. Filtered zein (10 mg/mL) dissolved in 80% ethanol was added dropwise to the solution to obtain different ratios of CL113: zein (1:0, 1:0.5, 1:1 and 1:2) and stirred for 30 min, respectively. LKP/IPP CZ NP were isolated by ultracentrifugation (Centriplus YM-30, MWCO of 30 kDa, Millipore, Darmstadt, Germany). Ethanol was removed using rotary evaporation (175 mbar at 40 °C) and IPP/LKP CZ NP were further analysed. To further maximise the loading of IPP and LKP, concentrations from 100 µg/mL to 700 µg/mL were formulated before zein coating and later lyophilised at concentrations of trehalose up to 5% *w*/*v*. Physiochemical characterisation and stability analysis were performed to determine the optimum concentration of trehalose.

### 4.4. Physicochemical Characterisation of CZ NP

The nanoparticle size and electrophoretic mobility were measured in folded capillary cells using a Nanosizer ZS fitted with a 633 nm laser (Malvern Instruments Ltd., Malvern, UK). Each analysis was carried out in triplicate at 25 °C with the equilibration time set to 2 min. The morphology of CZ NP was analysed using scanning electron microscopy (SEM) (Hitachi SU6600 FESEM), at an accelerating voltage of 20 kV using the secondary electron detector. The freeze dried NP (0.5 mg) were dispersed in deionised water (10 mL) and tip sonicated (Branson Ultrasonics; Ultrasonic processor VCX-750 W, Wilmington, NC, USA) for 4 min (25 s pulse on and off). One drop of the dispersion containing zein was placed on a silicon wafer and dried at room temperature. This was sputter coated with 4 nm Au/Pd prior to imaging. Fourier transform infrared spectroscopy (FTIR) was performed using a Perkin Elmer Spotlight 400 Series Spectrometer. FTIR spectra of zein, CL113, IPP/LKP NP and CZ NP were obtained in the spectral range 650 to 4000 cm^−1^. NP samples were stored at −80 °C in glass vials and then lyophilised prior to analysis using a Labconco FreeZone 6 Litre Benchtop Freeze Dry System.

### 4.5. Determination of Encapsulation Efficiency and Loading Capacity

The encapsulation efficiency (EE%) and loading capacity (LC%) of NPs were determined using an indirect method [69]. In brief, the supernatant was measured for the content of LKP or IPP by RP-HPLC performed on a Waters 1525 pump (Waters, Milford, MA, USA) with a Photo Diode Array detector 2487 (Waters) using a Luna C18 column (5 µm, 250 mm × 4.6 mm, Phenomenex). LKP analytes were detected at a wavelength of λ_max_ = 220 nm. The column was eluted at a flow rate of 1 mL min^−1^ with an isocratic system (15% Acetonitrile, 0.05% TFA, 84.95% water) and 10 µL injection volume. For IPP, analytes were detected at a wavelength of λ_max_ = 215 nm, eluted at a flow rate of 0.8 mL min^−1^ with an isocratic system (20% Acetonitrile, 0.05% TFA, 79.95% water) and 5 µL injection volume. The bioactive EE% and LC% were calculated using the following equations:

(1) $$\mathrm{EE}\%\;=\frac{\left(\mathrm{Total}\;\mathrm{amount}\;\mathrm{Peptide}\;-\;\mathrm{free}\;\mathrm{Peptide}\;\mathrm{in}\;\mathrm{supernatant}\right)}{\mathrm{Total}\;\mathrm{amount}\;\mathrm{of}\;\mathrm{Peptide}}\;\times 100$$

(2) $$\mathrm{LC}\%\;=\frac{\left(\mathrm{Total}\;\mathrm{amount}\;\mathrm{Peptide}\;\mathrm{added}\;-\;\mathrm{free}\;\mathrm{Peptide}\;\mathrm{in}\;\mathrm{supernatant}\right)}{\mathrm{Nanoparticle}\;\mathrm{weight}}\;\times 100$$

### 4.6. ACE Inhibition Assessment of CZ NP

ACE inhibition IC_50_ values were determined for IPP and LKP at concentrations 0.001–50 µM, using an ACE inhibition assay with Hippuryl-L-histidyl-L-Leucine (HHL) as substrate. HHL (5 mM) was dissolved in pH 8.3 buffer (0.1 M borate buffer in 0.3 M NaCL). An amount of 100 µL substrate solution and 25 µL inhibitor (peptide or peptide loaded NP) were incubated for 10 min at 37 °C. Then, 10 µL ACE solution (100 mU/mL) was added and after 30 min reaction was terminated using 100 µL of 1 M HCL. HPLC was performed using a C8 column (2.7 µm, 3.0 × 100 mm, Agilent Technologies UK & Ireland Ltd., Cork, Ireland) at a wavelength of 228 nm. An isocratic method was used at a flow rate of 0.4 mL min^−1^, 25% acetonitrile, 0.1% TFA in 74.9% water for 5 min. Captopril (10 µM) and unloaded NP were used as controls. The % of ACE activity was determined using the Hill Slope equation (Prism 5, GrapPad^®^ Software Inc., San Diego, CA, USA). CZ NP (40 mg) were lyophilised, resuspended in 20 mL water and sonicated for 8 min at 40% amplitude. The bioactivity of the released peptides was assessed to confirm lack degradation during formulation. Calculations of ACE inhibitions were based on the EE% of loaded formulations and a LC% (95 ± 1%) after lyophilisation.

### 4.7. Stability of CZ NP after Freeze Drying

The change in particle size and zeta potential was measured for CZ NP after lyophilisation; the least stable ratio (CL113: TPP) was chosen (ratio 6) to observe capacity to re-suspend after freeze drying. Trehalose in concentrations up to 5% *w*/*v* was added to the NP and lyophilised for up to 72 h to predict the minimum amount of trehalose needed to maintain the physicochemical characteristics of the CZ NP after drying. Dried unloaded CZ NP were resuspended in PBS (pH 7.4) and the particle size was measured using dynamic laser scattering to determine the aggregation properties of CZ NP. To assess the minimum concentration of trehalose needed in the formulation and its effect in the final bioactive loading, an inverse calibration was conducted. The particle size dependence on trehalose addition was modelled using a logistic model assuming that a critical concentration of trehalose addition was needed to protect the surface of the particles from agglomeration [70].

(3) $$\mathrm{Size}={\mathrm{Size}}_{0}+\frac{\left({\mathrm{Size}}_{1}-{\mathrm{Size}}_{0}\right)}{1+e^{\frac{\left(x_{\mathrm{mid}}-\mathrm{Trehalose}\right)}{\mathrm{scal}}}}$$

where Size_0_ is the particle size without addition of trehalose, Size_1_ is the particle size of the formulation completely protected by trehalose, x_mid_ is the amount of trehalose necessary to decrease the particle size halfway between Size_0_ and Size_1_ and scal is a scale parameter for the transition.

The zeta potential dependence on trehalose addition was modelled using a Michaelis-Menten model, assuming that trehalose had a catalytic effect on reducing the rate of agglomeration [71]:

(4) $$\mathrm{ZP}=\frac{{\mathrm{ZP}}_{\max}\mathrm{Trehalose}}{k+\mathrm{Trehalose}}$$

where ZP is the zeta potential, ZP_max_ is the maximum zeta potential and k is the trehalose concentration that will result in an increase of half of the ZP_max_.

The R language library [72] was used to fit the two models to the data and to produce inverse calibration predictions.

### 4.8. Cytotoxicity Assessment

Caco-2 and HepG2 cells were seeded at a cell density of 2 × 10^4^ cells/well in DMEM and EMEM, respectively. Both cells were incubated for 24 h at 37 °C in a humidified incubator with 5% CO_2_ and 95% O_2_. Test samples were incubated on Caco-2 cells for 4 h to mimic intestinal exposure [13], and HepG2 cells for 72 h to mimic liver exposure [13]. Test samples included LKP/IPP, loaded CL113 NP and loaded CZ NP (in serum free medium), at concentrations of 1, 5 and 10 mM. Triton X-100™ (0.05%) was used as a positive control. After exposure, treatments were removed and replaced with MTS (3-(4,5-dimethylthiazol-2-yl)-5-(3-carboxymethoxyphenyl)-2-(4-sulfophenyl)-2H tetrazolium. Optical density (OD) was measured at 490 nm. For NP formulations, medium without FBS was used to reduce aggregation via protein adsorption. Each value presented was normalised to untreated control and calculated from three separate experiments, each of which included six replicates.

### 4.9. In Vitro Dissolution Studies

The release of the peptide from zein coated NP was studied using a dialysis method similar to Bagre et al. but with slight modifications [73]. Simulated fluids were prepared in accordance with British Pharmacopeia 2016 [74], dissolution for delayed release of solid dosage forms (Method B), with the additional use of a dialysis membrane. Exposure for 2 h in simulated gastric fluid (SGF), followed by 4 h in simulated intestinal fluid (SIF), was performed for peptide loaded NP and loaded CZ NP. Then, 5 mL freeze-dried CZ NP in 2.5% trehalose were placed in a cellulose dialysis bag (cut-off 10 kDa, Spectra-Por^®^ Float-A-Lyzer^®^ G2). The dialysis bag was placed in the dissolution medium (50 mL), set at 100 rpm and maintained at 37 °C using a thermostatic shaker. Samples were withdrawn at different time intervals; 1 mL of release was removed and replaced with 1 mL simulated buffer at each time point. The samples were measured using RP-HPLC. The RP-HPLC analysis was performed on a Waters 1525 pump (Waters, Milford, MA, USA) with a Photo Diode Array detector 2487 (Waters) using a Luna C18 column 5 μm, 250 mm × 4.6 mm (Phenomenex, Chesire, UK). Analytes were detected at the wavelength of λ_max_ = 220 nm (LKP) and 215 nm (IPP). For LKP; the column was eluted at a flow rate of 1 mL min^−1^ with an isocratic system (15% Acetonitrile, 0.05% TFA in 84.95% water) and 10 μL injection volume. For IPP; the column was eluted at a flow rate of 0.8 mL min^−1^ with an isocratic system (20% Acetonitrile, 0.05% TFA in 79.95% water) and 5 μL injection volume.

### 4.10. Release Kinetics

The release kinetics from CZ NP in the SGF and SIF sequential controlled release experiments were fitted using the Peppas swellable model (Equations (5) and (6)). The model indicated diffusion-controlled and relaxation-controlled phenomenon according to the Higuchi equation for glassy polymers [75], previously used for LKP and IPP NP.

Peppas swellable for the SGF:

(5) $$\frac{M_{t}}{M_{\infty }}={\mathrm{ks}}_{1}*\left(\sqrt{\mathrm{time}}\right)+{\mathrm{ks}}_{2}*\mathrm{time}$$

where ks_1_ and ks_2_ are diffusive (Higuchi) and relaxation rate constants.

Peppas swellable for the SIF:

(6) $$\frac{M_{t}}{M_{\infty }}-\frac{M_{120}}{M_{\infty }}={\mathrm{ki}}_{1}*\left(\sqrt{\mathrm{time}-120}\right)+{\mathrm{ki}}_{2}*\left(\mathrm{time}-120\right)$$

where M_120_ is the predicted diffused mass at the time of changing from SGF to SIF (120 min), ki_1_ and ki_2_ are diffusive and relaxation rate constants. Nonlinear regression methods used for CZ NP were implemented using R software [72].

### 4.11. In Vivo Pharmacodynamic Studies

Male SHR (16-wk old SHR/NCrl; Charles River, Germany), weighing 260–320 g body weight and with SBP of over 170 mmHg, were randomised into groups (6 rats per treatment). Treatments were dissolved in Dulbecco’s phosphate-buffered saline (DPBS). IPP and LKP (10 mg/kg), IPP CZ NP and LKP CZ NP (10 mg/kg entrapped peptide), captopril (5 mg/kg) as positive control, and the negative control rats received the same volume DPBS. Following a single administration of treatments by oral gavage, the effects of treatments on SBP were compared. SBP was measured by tail-cuff plethysmography using the CODA^®^ Mouse Rat Tail Cuff Blood Pressure System (Kent Scientific, Torrington, CT, USA) at 0, 1, 2, 3, 4, 6, and 8 h (post oral administration of treatments) after warming the rat in a warming chamber kept at 34 °C for 5 min. Changes in SBP (Δ SBP) were calculated by setting untreated basal readings as zero using Prism-S^®^ software.

## 5. Conclusions

CZ NP loaded with the antihypertensive tripeptides IPP or LKP were successfully prepared via ionotropic gelation. The optimal IPP/LKP CZ NP showed desirable pharmaceutical properties for oral delivery through a systematic process-parameter investigation, including optimal size, high EE% and sustained release. The incorporation of zein as a secondary coating excipient significantly improved the encapsulation efficiency, resulting in spheroidal nanoparticles with a sustained delivery mechanism of action suitable for oral delivery. The oral delivery of peptides is often limited; however, this study showed that with the addition of zein, a delivery system with high potential for oral delivery can be achieved. In SHR studies, CZ NP exhibited an enhanced antihypertensive effect of both IPP and LKP with a longer therapeutic effect compared to free peptides and the data was similar to captopril. Oral administration of IPP/LKP CZ NP might be a potential strategy for hypertension treatment in the future.

## Figures and Tables

**Table 1:** Physicochemical characteristics of loaded peptide nanoparticles at optimised ratio of CL113: TPP.

Peptide	Peptide Load (µg/mL)	CL113 (mg/mL)	TPP (mg/mL)	Ratio (CL113:TPP)	Particle Size (nm)	ZP (mV)	EE (%)	LC (%)
LKP	100	1.58	0.27	6:1	166 ± 14	32 ± 2	65 ± 3	3 ± 0.5
IPP	100	1.45	0.40	4:1	136 ± 23	29 ± 2	41 ± 5	2 ± 0.8

**Table 2:** Physicochemical characteristics of LKP/IPP CZ NP at the optimal ratios of CL113: TPP and zein.

Peptide	Peptide Load (µg/mL)	CL113 (mg/mL)	TPP (mg/mL)	Zein (mg/mL)	Particle Size (nm)	ZP (mV)	EE (%)	LC (%)
LKP	100	1.58	0.27	1.58	301 ± 16	41 ± 1	80 ± 1	12.3 ± 0.3
IPP	100	1.45	0.40	1.45	223 ± 9	46 ± 2	84 ± 3	11.6 ± 0.5

**Table 3:** Percentage of ACE inhibition of free IPP/LKP, uncoated NP and CZ NP at peptide concentrations of 10 µM. Captopril (10 µM) and unloaded NP were used as positive and negative controls, respectively. Data are expressed as a mean ± SD of n = 6.

	ACE Inhibition (%)		
Concentrations	Native	CL113 NP	CZ NP
IPP (10 µM)	79 ± 2	81 ± 3	83 ± 9
LKP (10 µM)	80 ± 1	83 ± 2	84 ± 11
**Controls**			
Captopril (10 µM)	86 ± 4	-	-
Unloaded NP	-	5 ± 2	6 ± 5

**Table 4:** Swellable Peppas model parameters for kinetic release studies of CL113 nanoparticles loaded with LKP and IPP. Where IPP and LKP both represent uncoated control formulations in *ks* (stomach compartment) and *ki* (intestinal compartment) divided into diffusion and relaxation mechanisms (1 and 2). All parameters listed were statistically significant (<0.05).

Coefficients	Estimated	t-Value	*p*-Value
**IPP NP**			
*ks*_1_	14.7 ± 0.5	27.3	0.000
*ks*_2_	−0.7 ± 0.1	−12.5	0.000
*ki*_1_	1.4 ± 0.2	9.1	0.000
**LKP NP**			
*ks*_1_	−2.3 ± 0.6	−3.8	0.000
*ks*_2_	0.2 ± 0.1	3.1	0.002
*ki*_1_	1.4 ± 0.2	9.1	0.000
**CZ NP (IPP and LKP)**			
*ks*_2_-*ks*_2.(*CZ*)_	0.2 ± 0.1	4.4	0.000
*ki*_1_-*ki*_1.(*CZ*)_	3.7 ± 0.2	24.6	0.000

**Table 5:** Change in SBP of SHRs after single oral doses of various treatments.

Time (Hours)	PBS	Captopril	Unloaded NP	IPP	IPP-NP	LKP	LKP-NP
0	−1.7 ± 1.8	−1.2 ± 1.9	−0.1 ± 2.7	−2.9 ± 2.7	−0.3 ± 2.4	−3.4 ± 1.8	−4.9 ± 3.2
1	−4.7 ± 2.2	−14 ± 2.4 ***	−6.7 ± 1.2	−12.6 ± 1.8 *	−4.4 ± 1.8	−12.8 ± 2.2 *	−2.5 ± 2.3
2	−6.7 ± 2.2	−27 ± 1.6 ***	−5.4 ± 1.8	−22.5 ± 1.9 ***	−21.8 ± 1.6 ***	−21.2 ± 2.9 ***	−23.1 ± 2.8 ***
3	−6.6 ± 1.7	−28.2 ± 1.6 ***	−5.8 ± 2.5	−26.5 ± 2.6 ***	−23.5 ± 1.4 ***	26.5 ± 1.9 ***	−26 ± 2.4 ***
4	−4.8 ± 1.8	−29.2 ± 2.9 ***	−4.7 ± 1.3	−16.6 ± 3.1 **	−19.8 ± 1.8 ***	−16.5 ± 2.4 ***	−19.6 ± 3.5 ***
6	−6.8 ± 1.3	−21.9 ± 2.5 ***	−4.2 ± 1.2	−15.1 ± 4.3	−17.4 ± 1.1 **	−9.2 ± 1.7	−19.4 ± 1.3 ***
8	−6.7 ± 1.1	−17.5 ± 2.2 *	−5.7 ± 1.6	−7.8 ± 2.6	−15.7 ± 1.8 *	−3.6 ± 1.2	−16.3 ± 1.1 *

*** *p* < 0.001, ** *p* < 0.01, * *p* < 0.05 compared to PBS control. Two-way ANOVA with Bonferonni’s post hoc test multiple comparison; each value represents the mean ± SEM with n = 6 independent animals.
